# Systemic Bisperoxovanadium Activates Akt/mTOR, Reduces Autophagy, and Enhances Recovery following Cervical Spinal Cord Injury

**DOI:** 10.1371/journal.pone.0030012

**Published:** 2012-01-10

**Authors:** Chandler L. Walker, Melissa J. Walker, Nai-Kui Liu, Emelie C. Risberg, Xiang Gao, Jinhui Chen, Xiao-Ming Xu

**Affiliations:** 1 Department of Anatomy and Cell Biology, Stark Neurosciences Research Institute, Indiana University School of Medicine, Indianapolis, Indiana, United States of America; 2 Medical Neuroscience Graduate Program, Stark Neurosciences Research Institute, Indiana University School of Medicine, Indianapolis, Indiana, United States of America; 3 Spinal Cord and Brain Injury Research Group, Stark Neurosciences Research Institute, Indiana University School of Medicine, Indianapolis, Indiana, United States of America; Emory University, United States of America

## Abstract

Secondary damage following primary spinal cord injury extends pathology beyond the site of initial trauma, and effective management is imperative for maximizing anatomical and functional recovery. Bisperoxovanadium compounds have proven neuroprotective effects in several central nervous system injury/disease models, however, no mechanism has been linked to such neuroprotection from bisperoxovanadium treatment following spinal trauma. The goal of this study was to assess acute bisperoxovanadium treatment effects on neuroprotection and functional recovery following cervical unilateral contusive spinal cord injury, and investigate a potential mechanism of the compound's action. Two experimental groups of rats were established to 1) assess twice-daily 7 day treatment of the compound, potassium bisperoxo (picolinato) vanadium, on long-term recovery of skilled forelimb activity using a novel food manipulation test, and neuroprotection 6 weeks following injury and 2) elucidate an acute mechanistic link for the action of the drug post-injury. Immunofluorescence and Western blotting were performed to assess cellular signaling 1 day following SCI, and histochemistry and forelimb functional analysis were utilized to assess neuroprotection and recovery 6 weeks after injury. Bisperoxovanadium promoted significant neuroprotection through reduced motorneuron death, increased tissue sparing, and minimized cavity formation in rats. Enhanced forelimb functional ability during a treat-eating assessment was also observed. Additionally, bisperoxovanadium significantly enhanced downstream Akt and mammalian target of rapamycin signaling and reduced autophagic activity, suggesting inhibition of the phosphatase and tensin homologue deleted on chromosome ten as a potential mechanism of bisperoxovanadium action following traumatic spinal cord injury. Overall, this study demonstrates the efficacy of a clinically applicable pharmacological therapy for rapid initiation of neuroprotection post-spinal cord injury, and sheds light on the signaling involved in its action.

## Introduction

Few effective treatments exist for traumatic spinal cord injury (SCI). Difficulties in therapeutic development derive from complex temporo-spatial management of two pathological phases of SCI: primary mechanical injury, and secondary damage instigated by, and succeeding the initial trauma. Secondary injury processes including inflammation, vascular and axonal disruption, excitotoxicity, and edema exacerbate the primary damage [1], [2] and as such, effective control over secondary injury is essential for limiting physical and functional deficits endured after SCI. Following central nervous system (CNS) trauma, rapid cellular necrosis occurs at the injury epicenter, while delayed onset of apoptosis and macroautophagy spread out into the lesion penumbra [3], contributing to secondary cell death [4], [5]. These processes are regulated, at least in part, by phosphatidylinositol-3-kinase (PI3K)/Akt signaling, with downstream mammalian target of rapamycin (mTOR) protein complexes involved in promoting cell survival and growth. Upon PI3K activation, the pro-survival kinase, Akt, can be phosphorylated and activated by 3′-phosphoinositide-dependent kinases (PDKs) 1 & 2 [6], [7], activating or inhibiting downstream effectors including mTOR [8], BAD [9], glycogen synthase kinase 3β (GSK3β) [10], and MDM2 [11]. It is generally accepted that activation of this pathway is beneficial for preventing cell death and enhancing cellular processes associated with growth and proliferation. Following CNS injury, however, many questions remain concerning the influence of this signaling pathway, and the cell-specific outcomes such activation imparts in response to the insult. Following SCI, Akt phosphorylation decreases at and around the injury epicenter, while a PI3K-mediated upregulation occurs in neurons peripheral to the injury core [12]. This phenomenon is also observed following focal ischemic brain injury, and is considered an endogenous neuroprotective response of surrounding neurons [13].

Under normal conditions, downstream effectors such as mTOR are relatively inactive in spinal cord neurons [14]. The PI3K antagonist and phosphatase and tensin homologue deleted on chromosome ten (PTEN), however, is highly expressed in adult CNS neurons [15], [16]. PTEN's negative regulation of PI3K, and thus its over-activity, is important for cell cycle regulation, cellular turnover, and cell motility [17]. Such regulation results from PTEN's key function as a phosphatidylinositol (3,4,5)-trisphosphate (PIP_3_) lipid phosphatase, opposing PI3K's activation of Akt through removal of a phosphate from PIP_3_ to form phosphatidylinositol (4,5)-bisphosphate (PIP_2_). The loss of PTEN or its function is beneficial following CNS injury and disease, promoting axonal regrowth following SCI [18], reducing motor neuron atrophy [19], and enhancing neural survival [20], [21]. Increased downstream PI3K/Akt signaling activity is implicated in these effects. Such evidence suggests a potential alteration in PTEN expression or activity following SCI. Small-molecule protein tyrosine phosphatase (PTP) inhibitors, bisperoxovanadium (bpV) compounds, are known to promote neuroprotection following thoracic SCI [22], and Parkinsonian neurodegeneration [23], though no specific cellular signaling mechanism was investigated in these reports. bpV compounds specifically and potently inhibit PTEN activity *in vitro* [24], and administration *in vivo* promotes neuroprotection in various CNS injury models through activation of the PI3K/Akt pathway [25]–[27]. Akt activity also negatively influences retrograde axonal degradation through inhibition of macroautophagy following axotomy [28], a result potentially promoted through inhibition of PTEN. Thus, targeting PTEN may be part of the mechanism of bpV-mediated neuroprotection observed following spinal trauma.

Pharmacological therapies for SCI are urgently needed; as such treatments are minimally invasive and can be applied rapidly, which are crucial for minimizing secondary damage. We hypothesized that acute bpV administration promotes neuroprotection and functional recovery, at least partly, through upregulation of PI3K/Akt activity and downregulation of autophagic processes following SCI. Using histologic and protein analyses, and application of a novel forelimb functional assessment, we examined potassium bisperoxo (picolinato) vanadium (bpV[pic])-mediated effects on neuroprotection, functional recovery, and the involvement of Akt/mTOR and autophagic activity following cervical unilateral contusive SCI. To our knowledge, these findings are the first to investigate a link between bpV-mediated neuroprotection and PI3K/Akt/mTOR signaling following SCI.

## Materials and Methods

### Animals and surgical procedures

Adult female Sprague-Dawley rats (200–250 g, Harlan) (*n* = 40) were housed in controlled conditions with a 12∶12 light∶dark schedule, and access to food and water *ad libitum*. Prior to surgery, the animals were anaesthetized intraperitoneally (IP) with ketamine (40 mg/kg)/xylazine (5 mg/kg), and either a laminectomy only, or unilateral cervical SCI was performed as modified from Gensel et al. [29]. Briefly, a customized vertebral stabilizer was set to support the 5^th^ cervical vertebrae and a partial unilateral laminectomy was performed to expose the right side of the spinal cord at the same level. With dura intact, the NYU MASCIS Impactor [30] (2.5 mm tip, 10 g weight, 12.5 mm height) was used to inflict a moderate unilateral injury [29]. Sham animals were excluded from injury. Following surgery, animals received an injection of 3 mL 0.9% saline subcutaneously for hydration and were placed in temperature-controlled housing overnight for monitoring recovery. All surgical and animal handling procedures were performed as approved under the Guide for the Care and Use of Laboratory Animals (National Research Council) and the Guidelines of the Indiana University School of Medicine Institutional Animal Care and Use Committee (Approval #0000003163).

### Drug Treatment

Two sets of rats were randomly designated for treatment with bpV(pic) (*n* = 14) (Enzo Life Sciences), with dosing modified from Zhang et al., [20] or vehicle (*n* = 12). After surgery and/or injury, these animals received an immediate IP injection of 400 ìg/kg bpV(pic) while a control group received 0.9% saline (vehicle). A third group served as a surgical control group (sham) also injected with vehicle according the prescribed dosing schedule (*n* = 14). Animals received a second dose of vehicle or bpV(pic) 2 hrs post-injury, and twice daily for 1 or 7 days thereafter.

### Western Blotting

Protein analysis followed procedures described previously [31]–[33] with modification. Briefly, a 10 mm spinal cord segment containing the injury epicenter was removed for protein extraction 24 hours following SCI. Equal protein concentration from each sample was loaded onto 8–10% polyacrylamide gels, separated by SDS-PAGE, and transferred to a polyvinylidene difluoride (PVDF) membrane by electrophoresis. The membranes were immunoblotted with the following primary antibodies: monoclonal mouse-anti PTEN (1∶200); monoclonal mouse anti-ribosomal protein S6 (1∶200) (Santa Cruz Biotechnologies); polyclonal rabbit anti-phospho Akt (Ser^473^) (1∶1,000), a marker used for assessing PI3K activation; monoclonal mouse anti-pan Akt (1∶1,500); polyclonal rabbit anti-phospho ribosomal protein S6 (Ser^235/236^) (1∶400) (Cell Signaling, Inc.), an indicator of mTOR activity; polyclonal rabbit anti-LC3B (1∶100; Abgent), to assess autophagy activity; monoclonal mouse anti-β-tubulin (1∶1000; Sigma-Aldrich) as a loading control. Membranes were incubated with a goat anti-rabbit or anti-mouse fluorescent secondary antibody, fluorescing at either 680 nm or 800 nm wavelength (1∶10,000; Rockland Immunochemicals, Inc.) and visualized using the Li-Cor Odyssey infrared imaging system and software (Version 1.2) per the manufacturer's instructions. Quantification of detected bands was performed by densitometry using ImageJ software (NIH).

### Histological Assessments

Six weeks post-injury, tissue was collected and processed as previously published [34]. In brief, a 1 cm segment of cervical cord including the injury epicenter was dissected and sectioned longitudinally in the horizontal plane or transversely at 20 ìm thickness on Superfrost Plus slides (Fisher Scientific) using a cryostat (Leica). Tissue was stained using cresyl violet acetate stain with eosin counterstaining. Lesion and cavity volume were calculated using Neurolucida software (MicroBrightfield, Inc.). Serial sections with an interval of 0.5 mm along the length of the cord were used for lesion volume measurement and calculation, spanning from tissue first showing identifiable lesion, to the last demonstrating such morphology. These same methods, equipment, and software were used to construct 3-dimensional representations of vehicle- and bpV-treated animal spinal cord tissue. Additionally, the same sections and staining used for assessing lesion, cavity, and spared tissue volume were used to quantify the number of motorneurons throughout the specified spinal cord distance (3 mm rostral and caudal to the epicenter). To define a standard anatomical area for counting motorneurons, a horizontal line was drawn from one side of the transverse section to the other passing through the central canal. All clearly identifiable ipsilateral motorneurons ventral to this line exhibiting visible nuclei in plane, and dark, evenly distributed cresyl violet staining, were manually quantified using ImageJ software.

### Vascular Quantification

Six weeks post-injury, spinal tissue sections 2 mm rostral and caudal, as well as from the epicenter of the lesion, were utilized for vascular quantification using a general rat endothelial cell marker, and calculation of the vessel-positive area in the ipsilateral and contralateral gray matter in vehicle- and bpV-treated animal groups. In brief, vessels in spinal tissue from bpV- and vehicle-treated animals were labeled via immunofluorescence with mouse anti-rat endothelial cell antigen-1 (RECA-1, 1∶200; ABD Serotec), a specific vascular marker, overnight at 4°C. The following day, the tissue was labeled with fluorescent secondary antibody, and high power images were acquired with an Olympus BX60 microscope and PictureFrame software (Optronics). RECA-1^+^ area was quantified using ImageJ software through methods modified from previous publication [22]. Briefly, the anatomically defined area of ipsilateral gray matter containing RECA-1^+^ vasculature was outlined and calculated per measured section. Only areas of gray matter clearly containing vasculature, as identified by RECA-1 labeling, were included in this assessment.

### Immunofluorescence Double Labeling

Immunofluorescence double labeling was performed as previously described [34]. Briefly, 1 day following SCI, 1 cm spinal tissue including the injury epicenter was removed after perfusion, and cryoprotected in 30% sucrose in 0.1 M PBS. Cord segments were sectioned and prepared for staining as described above, and incubated with the following primary antibodies: mouse and rabbit anti-PTEN (1∶100; Santa Cruz Biotechnologies), rabbit anti-phospho ribosomal protein S6 (Ser^235/236^) (1∶400) (Cell Signaling, Inc.), mouse anti-NeuN (1∶200; Chemicon), a marker for neurons; mouse anti-CC1 (APC-7, 1∶25; Calbiochem, Inc.), a marker for oligodendrocytes; mouse anti-OX42 (1∶50; Harlan Sera-lab), a marker for microglia; and rabbit and mouse anti-glial fibrillary acidic protein (GFAP, 1∶200; Sigma), a marker for astrocytes. The following day, the sections were incubated with rhodamine- or fluoroisothiocyanate-conjugated goat anti-rabbit or anti-mouse antibodies (1∶200; Jackson ImmunoResearch Lab). Sections were coverslip mounted with Fluoromount G combined with Hoechst 33342 (1∶100) for nuclear staining. Pre-immune serum was used as a control to confirm the specificity of the antibody. Images were obtained with a Nikon TE2000 laser scanning confocal microscope.

### Behavioral Testing

For functional assessment, cereal rings of uniform shape, size, and flavor were provided and arm joint articulation [35], movement, treat handling, and coordination were assessed while the animals ate the treat. Each rat was tested in a home cage, which promoted the most optimal conditions for assessing the animals' behavior and performance. Using an 8 point scale, with 8 indicating normal ability and 0 representing no ipsilateral forelimb treat contact ability, the animals were tested before SCI (baseline), and once per week for 6 weeks following injury, and were scored on three separate trials with the three scores averaged (Table S2). Treat manipulation was defined as apparent and consistent coordinated handling of the treat by both forelimbs during eating. The general scoring guidelines and categories for gauging functional improvement of the forelimb are provided in Tables S1 & S2. Visual examples of rats from each group eating the cereal treats are shown in Figure S1.

### Statistical Analysis

To determine significance between two groups, a two-tailed unpaired Student's *t*-test was used. Statistical significance between multiple groups was determined using a one-way ANOVA with Tukey's post-hoc analysis. All statistical values were calculated using GraphPad Prism 5.0 software (GraphPad, Inc.), with a *p* value<0.05 considered statistically significant.

## Results

### bpV(pic) promoted significant neuroprotection following cervical contusive SCI

**Figure 1:**
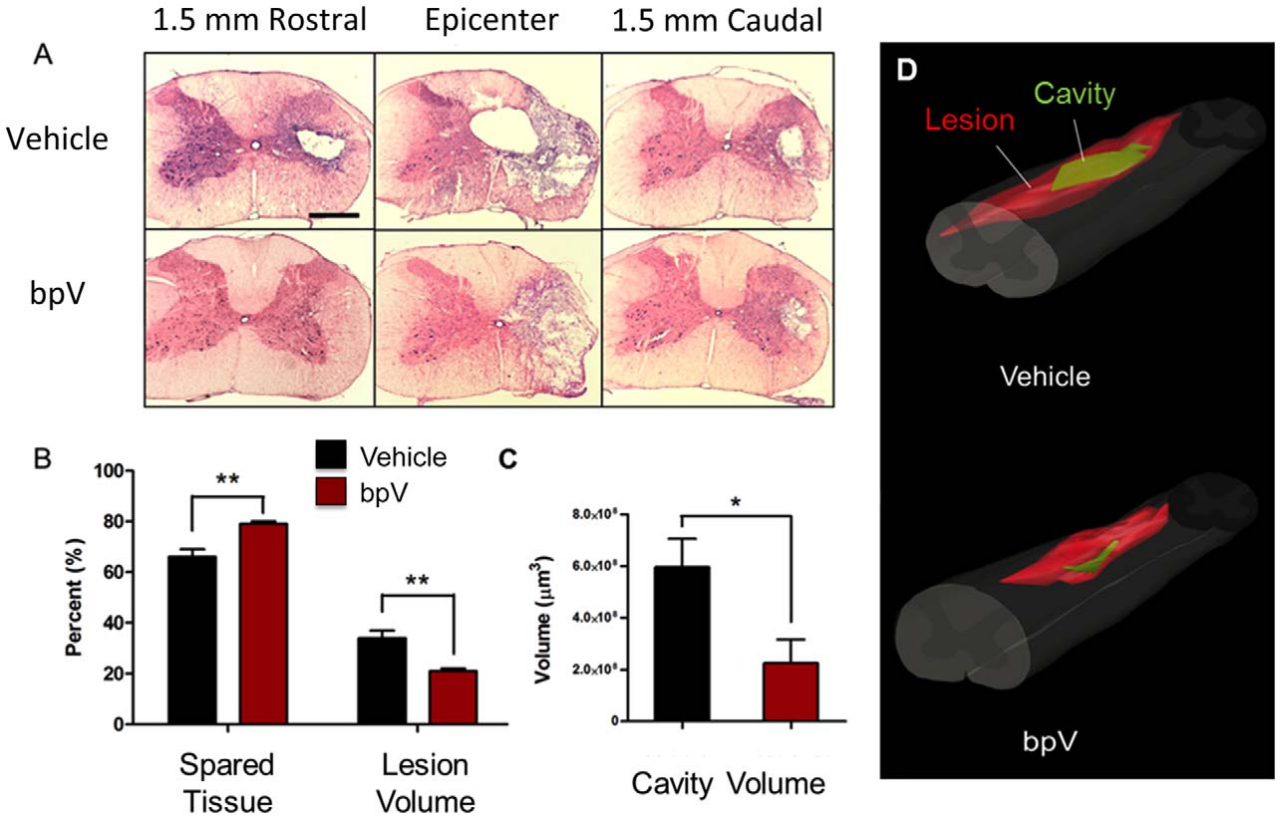
bpV(pic) reduces lesion size and cavitation following C5 hemicontusion SCI. A) Tissue extracted from rats 6 wks post-SCI demonstrate reduced lesion and cavitation through Nissl-eosin staining compared to vehicle treated animals. B & C) Graphical representation showing statistically significant reduction in lesion and cavity volumes, and increased spared tissue following bpV(pic) treatment. D) 3D-reconstruction from representative cases illustrating the neuroprotective effects of acute bpV(pic) therapy. **, *p*<0.01; *, *p*<0.05. *n* = 4−5. Error bars = SEM. Scale bar = 1 mm.

To assess whether bpV treatment promoted neuroprotection following injury, histological analysis was utilized. bpV-treated animals (*n* = 5) had significantly reduced ipsilateral lesion (21%±1.1 vs. 34%±3.0 SEM; *p*<0.01) and cavity volume (approx. 67% reduction; *p*<0.05) compared to vehicle-treated animals (*n* = 4) (Fig. 1A & B). Sham animals showed no sign of injury (*n* = 5). In comparison to the relative area of the ipsilateral cord, spared tissue was significantly increased in bpV-treated animals compared to vehicle controls (79%±1.1 vs. 66%±3.0 SEM; *p*<0.01). Additionally, bpV treatment greatly reduced the amount of cavitation typically observed in subacute to chronic phases after contusive SCI, as seen in the vehicle-treated group (Fig. 1C & D).

**Figure 2:**
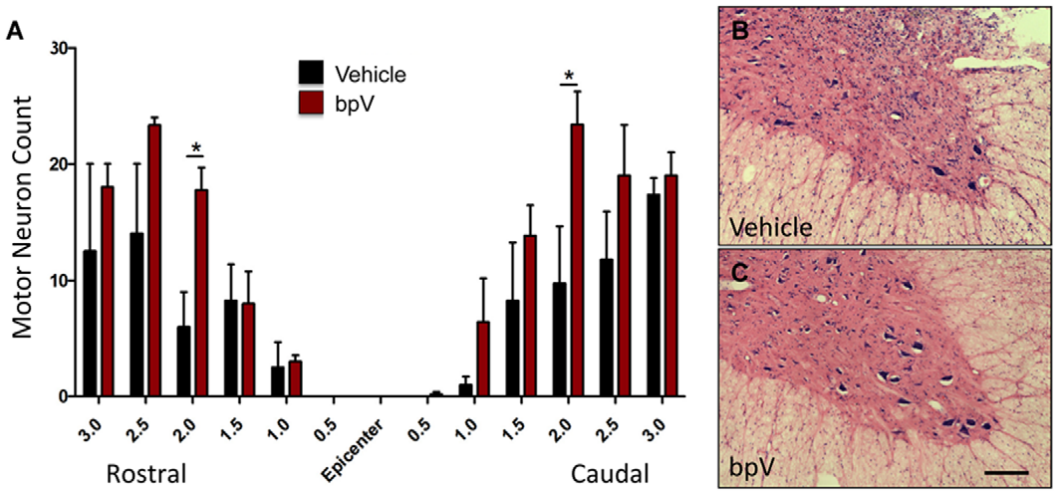
Acute bpV therapy reduces motor neuron loss following SCI. A) bpV(pic) treatment spared, on average, more ventral horn motor neurons than vehicle-treatment. Significantly more motor neurons were present in the ventral horn 2 mm rostral and caudal to the lesion epicenter of bpV(pic) treated animals. B & C) Photomicrographical representation showing Nissl-eosin stained ventral horns of spinal tissue extracted 6 weeks post-SCI. Sections shown are from 2 mm rostral to the epicenter and demonstrate the increase in motor neurons in bpV-treated animals over vehicle-treatment. *, *p*<0.05. *n* = 4−5. Error bars = SEM. Scale bar = 150 µm.

**Figure 3:**
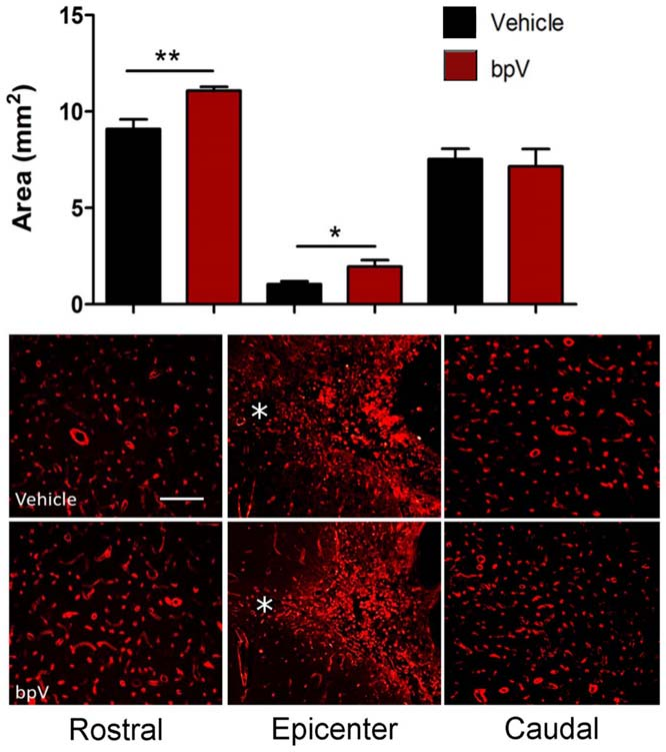
Significant increase in ipsilateral gray matter vasculature rostral and at the epicenter of the injury. bpV(pic)-treated animals demonstrated significantly increased vascular (RECA-1)-positive area in the ipsilateral gray matter 2 mm rostral and at the epicenter of the lesion 6 weeks post-SCI. * = central canal in photomicrographs. No significant difference was observed 2 mm caudal to the epicenter. **, *p*<0.01; *, *p*<0.05. *n* = 4−5. Error bars = SEM. Scale bar (Rostral & Caudal) = 100 µm; (Epicenter) = 150 µm.

Consistent with reduced lesion volume, bpV-treated animals exhibited a trend of increased ventral horn motorneurons near the injury epicenter than vehicle-treated animals at 6 weeks post-injury, with significantly increased motorneurons noted both 2 mm rostral and caudal to the injury epicenter (Fig. 2A). Representative photomicrographs demonstrating the difference in motorneuron number in the rostral ipsilateral ventral horn between untreated and bpV-treated animals are shown in Fig. 2B and C. As vascular sparing and integrity have been attributed to bpV-mediated neuroprotection following SCI, using methods of vascular quantification modified from previous publication [22], RECA-1^+^ vascular area (mm^2^) was assessed in anatomical regions of interest (gray matter) from transverse spinal cord sections 2 mm rostral and caudal, and at the injury epicenter (Fig. 3). Spinal cord RECA-1^+^ (vascular) gray matter area was significantly increased in bpV(pic)-treated rats compared to vehicle-treated animals (*p*<0.01) both rostral and at the epicenter of the injury (*p*<0.05). No difference in gray matter vascular area was observed caudal to the injury site (Fig. 3). The contralateral gray matter vascular area, as expected, did not exhibit differences between treatment groups rostral or caudal to the injury. However, significantly more vascular area was measured in the bpV-treated group within the contralateral gray tissue at the injury epicenter in comparison to the vehicle-treated group (*p*<0.05). This outcome was likely a result of neuroprotection mediated by treatment (data not shown).

### Skilled forelimb articulation and coordination improved following bpV(pic) treatment

**Figure 4:**
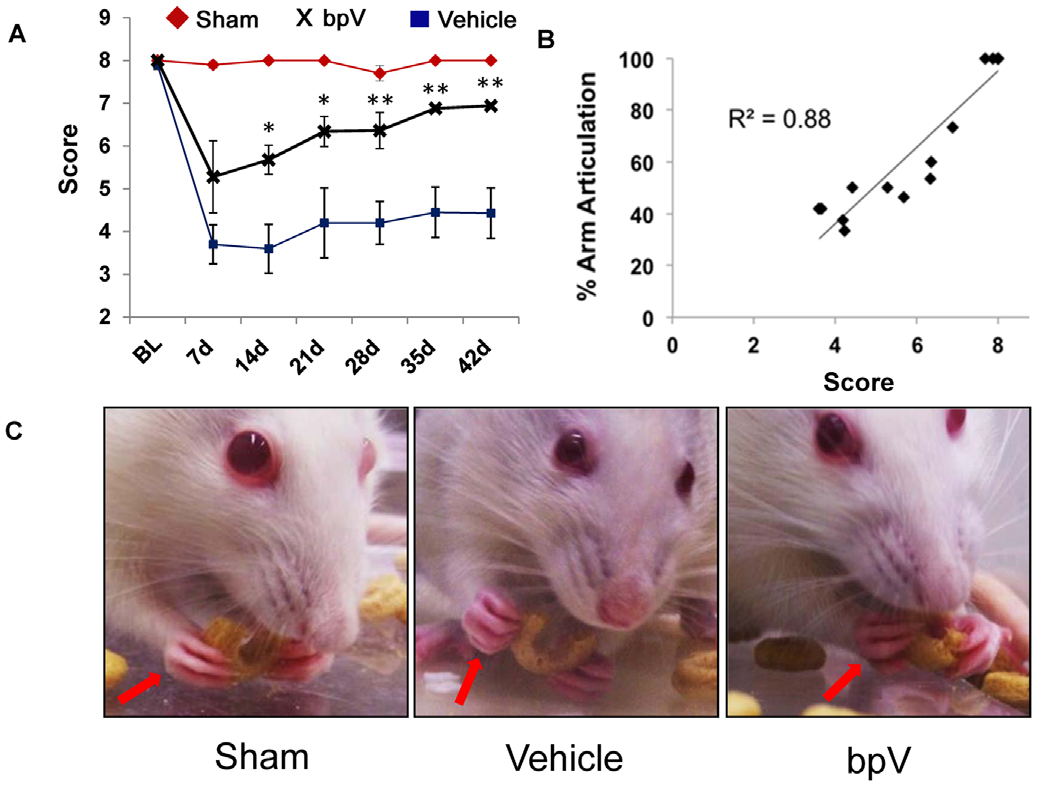
bpV-treatment enhances forelimb functional recovery. A) By 6 weeks post-injury, bpV(pic)-treated rats exhibited significantly enhanced forelimb function over vehicle-treated animals as scored using an 8-point treat manipulation behavioral scale (6.8 vs. 4.5). Sham animals scored at or near perfect (8 points) throughout the study. B) Testing scores showed a positive linear correlation with arm joint articulation ability (*R^2^* = 0.88). C) Images portraying a rat grasping and manipulating a flavored cereal ring, the treat used in this assessment. **, *p*<0.01; *, *p*<0.05. *n* = 4−5. Error bars = SEM.

To determine whether the observed neuroprotection mediated by bpV enhanced functional recovery, a novel forelimb behavioral assessment was used. During the 6 week behavior testing, sham animals (*n* = 5) achieved perfect scores (8 = maximum score) overall during testing, as expected (Fig. 4A). Animals treated with bpV (*n* = 5) recovered significant skilled forelimb function and coordination while eating cereal rings compared to vehicle-treated animals (*n* = 4) using our independently-developed forelimb coordination and manipulation test (Fig. 4A & C). bpV-treated animals averaged a score of 6.94±0.06, while vehicle-treated animals averaged 4.43±0.59 by the end of 6 weeks. Scores obtained during skilled forelimb use demonstrated a highly linear correlation with arm articulation ability (*R^2^* = 0.88; Fig. 4B), as scored as a subset from a forelimb locomotion test published and used by Martinez et al. [35].

### Acute cell-specific expression profiles of PTEN and phospho-S6 after cervical SCI

**Figure 5:**
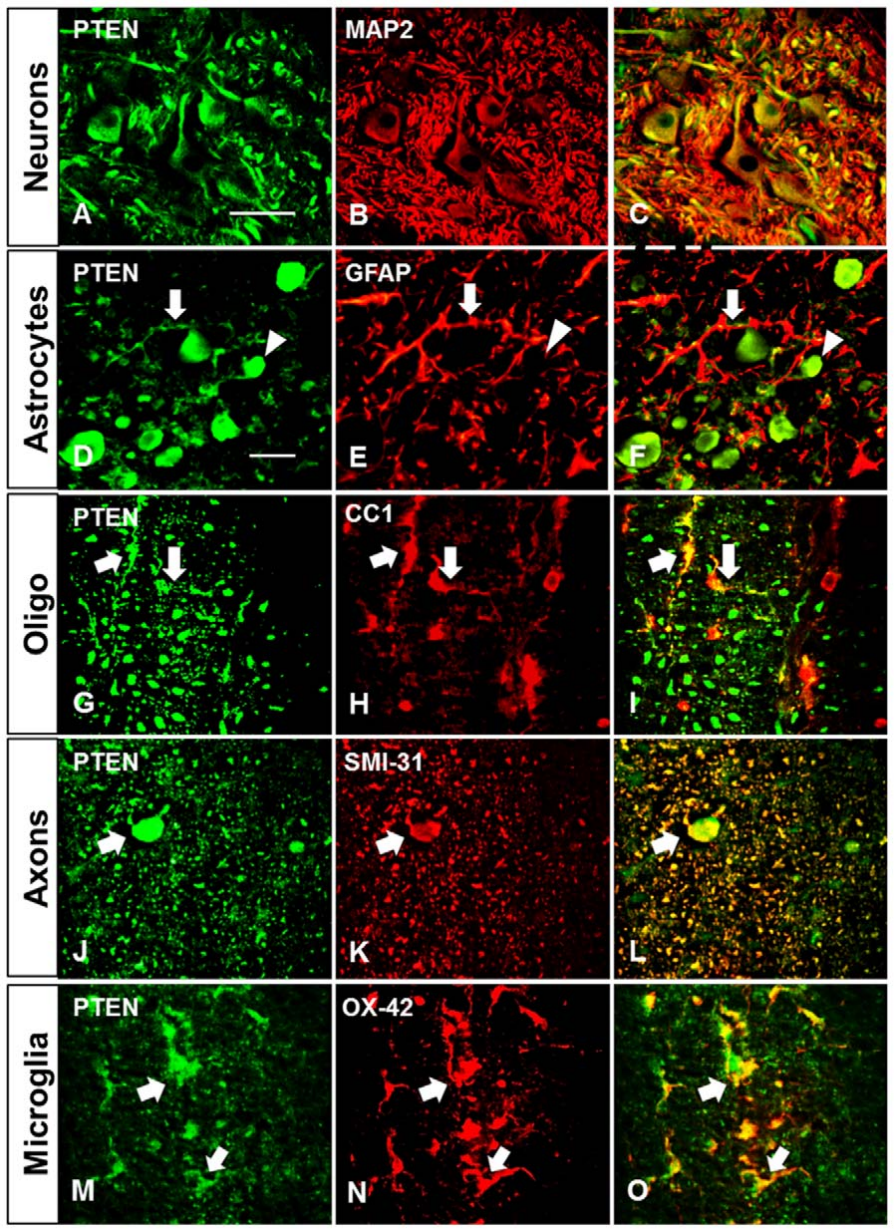
PTEN cellular localization following injury. A–C) PTEN highly colocalized to MAP2^+^ neurons however, PTEN only somewhat colocalized with D–F) GFAP^+^ hypertrophic astrocytes near the lesion (arrow), but highly localized to axons (arrowhead). G–I) CC-1^+^ oligodendrocytes, J–L) both smaller and swollen (arrow) degenerating axons within white matter tracts surrounding the injury, and M–O) OX-42^+^ reactive microglia also expressed considerable PTEN 1d following SCI. *n* = 3. Scale bar = 00 µm.

**Figure 6:**
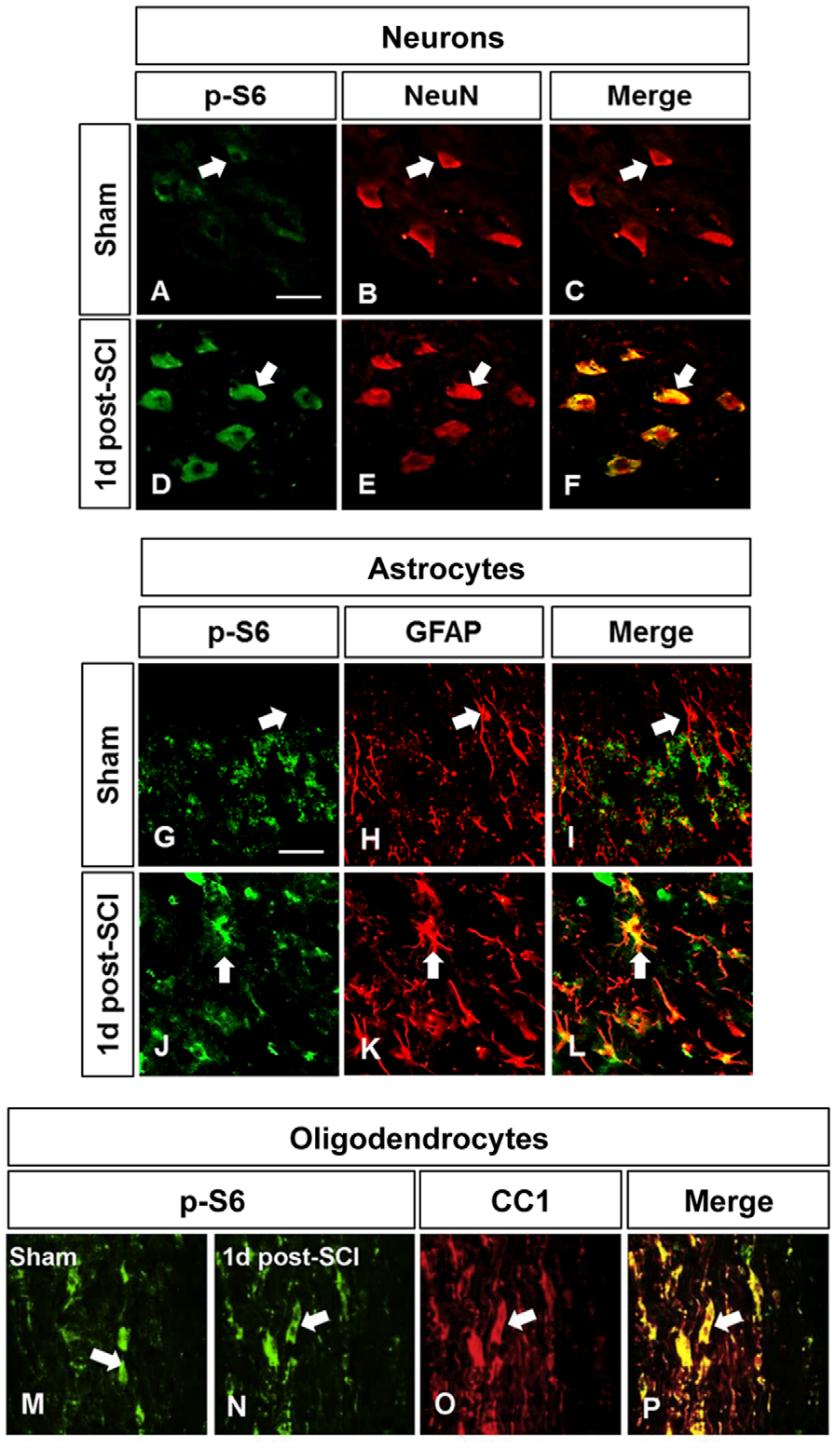
Phospho-S6 cellular localization following injury. Like PTEN, phospho-S6 (p-S6) colocalized with many cell and structure types 1d following SCI. p-S6 was expressed very abundantly in A–F) neurons, and G–L) hypertrophic astrocytes following SCI, as well as M–P) oligodendrocytes, as indicated by arrows. *n* = 3. Scale bar = 100 µm.

As we hypothesized that bpV may act through PTEN functional inhibition for enhancement of neuroprotection and recovery, immunofluorescence double-labeling of PTEN with various cell types and structures was performed for identification of potential cellular targets affected by bpV treatment (Fig. 5). PTEN was highly expressed in motorneurons (Fig. 5A–C), CC1^+^ oligodendrocytes (Fig. 5G–I), and OX-42^+^ reactive microglia (Fig. 5J–L) after injury, though some minor expression was seen in reactive astrocytes post-SCI (Fig. 5D–F). As PTEN inhibition promotes upregulation of PI3K/Akt/mTOR signaling, cell-specific co-localization with the mTOR activity marker phosphorylated ribosomal protein S6 (p-S6) was also performed. In general, p-S6 expression upregulation was observed in motorneurons (Fig. 6A–F) of injured animals, as well as hypertrophic reactive astrocytes (Fig. 6G–L) and oligodendrocytes (Fig. 6M–P) near the injury site.

### bpV(pic) significantly enhanced Akt/mTOR signaling, and reduced LC3 II/LC3 I protein ratio

**Figure 7:**
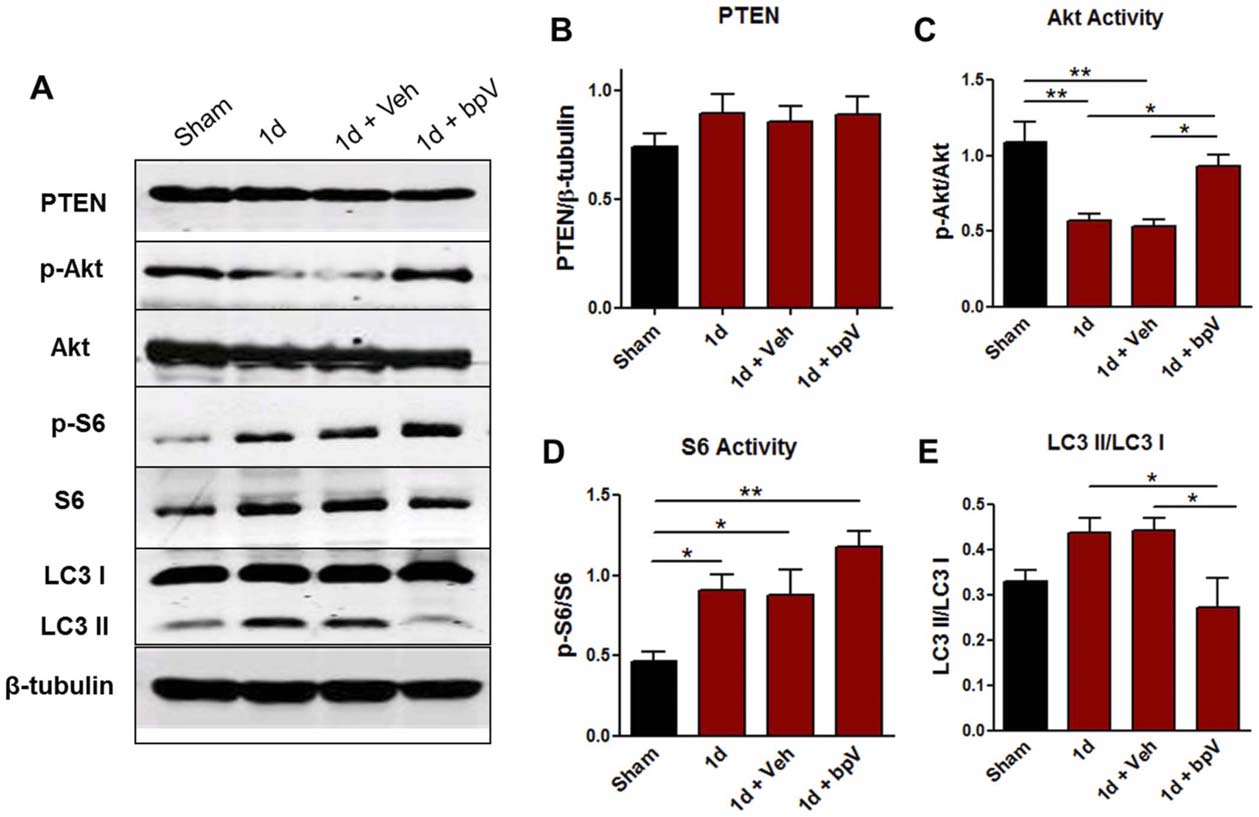
Effects of bpV(pic) on mTOR and autophagy-related protein analysis 1d post-SCI. A) Western blot profiles from tissue collected from experimental animals 1d post-SCI. B–E) Quantification of blots shown in A. B) Total PTEN protein expression does not significantly change following injury, though a mild increase in expression is observed. C) p-Akt levels significantly decrease following injury, and are significantly increased following bpV (pic) treatment. D) Downstream, p-S6 protein levels significantly increase following injury, and are enhanced further following bpV treatment. E) LC3 II ratio to LC3 I, an indicator of autophagic activity, is increased following injury, and is significantly reduced following bpV(pic) therapy. **, *p*<0.01; *, *p*<0.05. *n* = 4−5 per group. Error bars = SEM.

**Figure 8:**
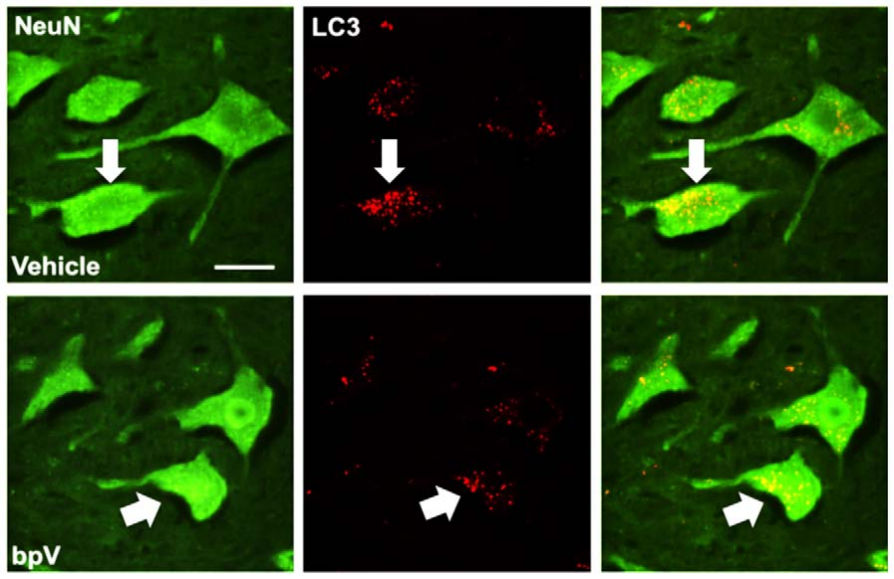
bpV(pic) reduces neuronal autophagosome aggregation. Vehicle-treated rats exhibited dense intracellular LC3-positive autophagosome aggregation in neurons (arrows). Treatment with bpV promoted a decrease in neuronal autophagosome clustering. *n* = 3. Scale bar = 50 µm.

For more accurate quantification of bpV's effects on PI3K/Akt/mTOR pathway-related protein expression changes in the cervical spinal cord, Western blot analysis was performed (Fig. 7A). PTEN expression levels were not significantly different between sham and 1d groups (*n* = 4−5, each), though a trend in increase was observed following injury (Fig. 7B). bpV treatment showed no significant effect on PTEN expression levels (*n* = 4−5). To determine whether SCI or bpV(pic) administration influenced PTEN's antagonism of PI3K and downstream mTOR activity, phosphorylated Akt (p-Akt) and p-S6 levels were assessed. 1 day following SCI, p-Akt significantly decreased in tissue including the injury epicenter (*p*<0.01), while p-S6 expression significantly increased (*p*<0.05). bpV-treated animals (*n* = 5) recovered p-Akt expression to near sham levels compared to injury-only and vehicle-treated animals (*n* = 5) (*p*<0.05), and elevated p-S6 expression further over sham (*p*<0.01), but not significantly over the other groups (Fig. 7C & D). Since mTOR is a known regulator of autophagy, autophagosome formation was measured by quantifying downstream microtubule-associated protein light chain 3 (LC3) protein levels. A band shift during SDS-PAGE blotting is indicative of LC3 I to LC3 II conversion, and thus an increase in autophagic activity [36]. The ratio of LC3 II to LC3 I increased in untreated animals 1d after injury, though this ratio significantly diminished to near sham levels of expression following bpV(pic) treatment (*p*<0.05), suggesting a reversal of autophagic activity upregulation seen following SCI (Figs. 7E & 8).

## Discussion

### Neuroprotection and functional recovery promoted by bpV

As predicted, by 6 weeks post-injury, bpV(pic) drastically reduced lesion and cavity volume, while increasing spared tissue, including vasculature, as well as skilled forelimb function over vehicle-treated control animals. The enhanced amount of vasculature-containing gray matter tissue could logically result from overall reduction of lesion and spread of secondary cell death observed following bpV therapy, or a stimulation of angiogenesis following this treatment, due to suppression of PTEN activity and subsequent upregulation of PI3K signaling [37]–[39]. A second explanation is that vascular sparing actually contributes to the overall neuroprotective effects, a hypothesis proposed by Nakashima et al. [22] after observing a similar phenomenon following bpV(phen) treatment for SCI. In support of this explanation, a recent study by Han et al. [40] showed that angiopoietin-1 and synthetic C16 peptide treatment spared vasculature by targeting the vascular endothelium, leading to reduced inflammation, diminished white matter loss, and functional recovery following SCI.

bpV also promoted significantly increased forelimb function compared to vehicle-treated animals beginning two weeks post-injury (*p*<0.05) with improvement observed until the end of testing (*p*<0.01). These results corresponded well to the significant neuroprotection observed in this study. Rats utilize proprioceptive ability to manipulate and eat through coordinated and articulated forelimb activity, allowing for a more sensitive assessment of the injured forelimb's functional ability. Also, the rats are innately motivated to eat the treats, requiring no training and allowing for easy repetition for multiple trials during each testing period without the need to fast the animals. A similar though separately developed test was recently reported by Irvine et al. [41], underscoring the need for reliable and sensitive assessments of overall fine forelimb function following cervical SCI. The details of their test also support the validity of our design, enhancing confidence that the increased performance during our forelimb assessment after bpV(pic) treatment is genuine.

### Linking bpV to Akt/mTOR signaling following cervical spinal injury

Though no significant PTEN expression change was observed, our results show that PTEN is highly expressed in neurons, CC1^+^ oligodendrocytes, and activated microglia 24 hours after injury. Reactive astrocytes expressed PTEN, albeit minimally, as well, after cervical SCI, suggesting that multiple cell types may be targets for bpV(pic) action. Overall p-Akt levels relative to pan-Akt decreased in cervical spinal tissue (Fig. 7A & C), suggesting that PTEN activity may increase post-SCI. Pilot data also suggested PTEN activity is likely increased through day 7 after SCI, with p-Akt remaining near levels shown for 1 day post-injury, and low-concentration bpV treatment through this period reversed p-Akt decrease (data not shown). This information influenced the experimental design and timeframe for delivering bpV in this study. Though we observed this reversible downregulation of Akt phosphorylation 1 day post-injury, an increase in downstream p-S6 was observed in neurons and increasingly hypertrophic astrocytes near the injury site (Fig. 6A–L), suggesting that neurons may upregulate mTOR activity for protein synthesis as a neuroprotective response, and glia for both neuroprotection and initiation of a reactive state following the injury, as discussed later in this section. Despite the increase in p-S6 seen in reactive astrocytes, no significant difference in GFAP reactivity, used as a general indicator of reactive astrogliosis, was observed between treated and non-treated animal groups 6 weeks post-SCI (Fig. S2). Interestingly, oligodendrocytes highly expressed p-S6 both pre- and post-injury (Fig. 6M–P), suggesting this mechanism may serve both a neuroprotective, as well as perhaps a myelin-related function in these cells. PTEN expression in oligodendrocytes is involved in maintenance of myelin and axonal integrity, though not for remyelination as demonstrated through PTEN knock-out experiments [42].

It is quite striking that neurons and glia drastically upregulate p-S6 following SCI, contradictory to expectations based on reports of benefits from PTEN inhibition following CNS injury. The discrepancy between Akt activity decrease and ribosomal protein activity increase may result from two different or interacting mechanisms via cross-talk between PI3K and mitogen-activated protein kinase (MAPK) signaling. It has been shown that following SCI, active phosphorylated extracellular signal-regulated kinase (Erk, MAPK) increased dramatically and rapidly [43]. Activated isoforms of Erk are known contributors to p70S6 kinase (p70S6K) upregulation along with mTOR [44], which could explain the significant increase in p-S6 following SCI, despite the down-regulation of p-Akt. Active Erk is also known to activate mTOR through upstream tuberous sclerosis complex 2 (TSC2) inhibition [45], and on p90 ribosomal protein kinase [46] potentially influencing p-S6 upregulation. Overall, the enhancement in p-Akt and p-S6 levels by bpV over non-treated controls provides a potential mechanism for the action of bpV(pic). However, mTOR-dependent and independent influences on neuroprotection and functional recovery are both possible explanations for results observed during this study.

Targeting translational upregulation through S6 activation may promote an endogenous neuroprotective response, contributing to various cellular activities triggered by the insult. This could also explain the upregulation of mTOR activity implicated in astrocyte reactivity witnessed here and elsewhere [47] in response to spinal injury. PTEN expression was not visually abundant in non-injured astrocytes, but was detected at low-levels in reactive astrocytes 1d post-injury, consistent with reports observing PTEN in astrocytes early in reactive astrogliosis, decreasing as reactivity peaks [48]. Though overall p-Akt levels decrease post-injury near the injury site, the increase in both p-Akt and p-S6 after bpV treatment suggests an enhanced benefit of upregulating both pathways, either through endogenous neuroprotective feedback, or another mechanism promoting amplification of p-S6 and other cell responses observed following SCI.

### Downregulation of autophagy following bpV treatment

Enhancement of endogenous cell survival mechanisms, such as autophagic activity, can rapidly stabilize the cell in response to hypoxic, ischemic, and inflammatory conditions. Autophagy is a normal cellular phenomenon required for degradation of organelles and proteins, and can be increased for energy acquisition when under cell or nutrient stress. Autophagy dysregulation, however, has been implicated as a cause of autophagic, or Type II programmed cell death following SCI [4], [5]. Contrasting the popular view that mTOR inhibits autophagy, p70S6K has been shown to enhance autophagic activity following mTOR activation [49]. However, this endogenous protection mechanism may not suffice to spare the cells from delayed death post-SCI, or perhaps the stimulation of autophagic activity proceeds from beneficial to detrimental, ultimately leading to programmed cell death. A continuum of biological processes within cells influenced by SCI including autophagic activity is likely in describing different stages leading to programmed cell death.

Our results suggest that bpV treatment may serve as an autophagic “switch” by forcing increased mTOR activation, and enhancing the inhibitory-actions of mTOR on autophagy. Overactivation of p70S6K is not associated with increased autophagic activity, and mTOR can act directly through regulation of autophagy-related proteins resulting in non-p70S6K dependent control of this process [49], supporting our findings and aiding in our interpretation of these results. If autophagic activity plays a role in neuronal programmed cell death, then bpV(pic) may act through PI3K and mTOR to downregulate this process, preventing the transition towards cell loss.

### Considerations for bpV as a therapy for SCI

Although bpV(pic) is an accepted inhibitor of PTEN activity, it may also exhibit other unknown functions that may or may not contribute to effects directly investigated in this study. Bisperoxovanadium compounds are inhibitors of PTP activity [50], some others of which may potentially be influenced by such therapy in addition to PTEN activity. One example involves indirect promotion of PTEN activity by bpV-mediated inhibition of the PTP, Src homology region 2 domain-containing phosphatase-1 (Shp-1), which is known to bind, dephosphorylate, and activate PTEN [51]. Src protein kinases serve several roles, one of which being involved in promoting cell survival through PI3K/Akt signaling [52]. Therefore, bpV could interfere with Src-related protein modulation of PTEN activity in addition to, or separate from, direct PTEN functional inhibition.

**Figure 9:**
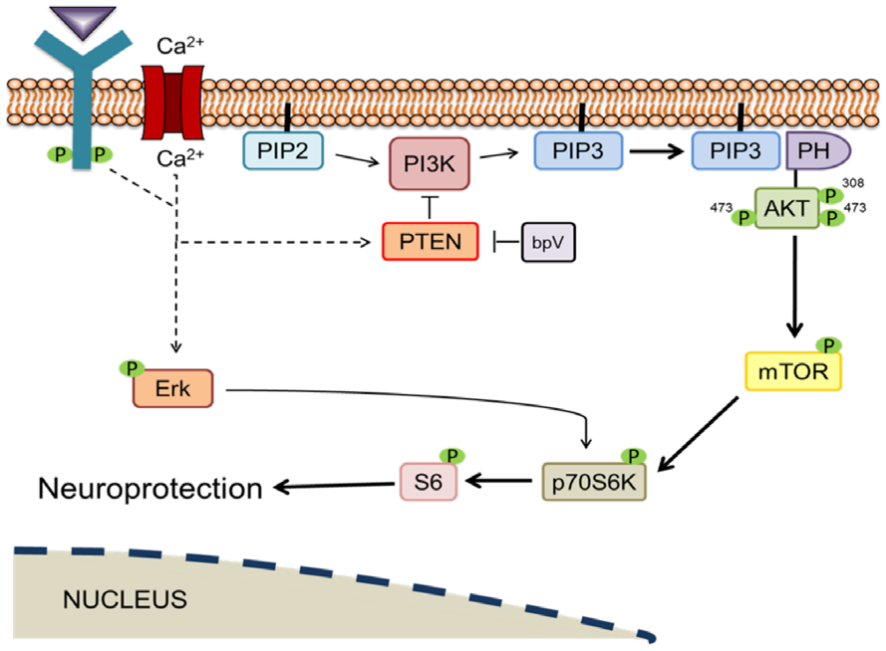
Potential mechanistic explanation for bpV(pic)-mediated neuroprotective effects. PTEN's phosphatase activity converts phosphatidylinositol (3,4,5)-trisphosphate (PIP_3_) into phosphatidylinositol (4,5)-bisphosphate (PIP_2_), thus inhibiting downstream Akt and mTOR signaling. PI3K converts PIP_2_ into PIP_3_, which can then activate Akt and mTOR, thus enhancing p-S6 expression and contributing to the decrease in cellular autophagic activity that may be involved in programmed cell death, and leading to neuroprotection.

PTEN/PI3K signaling is ubiquitous throughout the body; therefore, interfering with PTEN inhibition of PI3K signaling may have effects on unintended cellular targets. This is important in the spinal cord, in that glia and neurons respond differently to trauma. Astrocytes and microglia activation parallels the progression of secondary damage processes that promote neuronal death. For instance, it has been shown that astrocytes increasingly upregulate mTOR signaling [47] and downregulate PTEN expression [48] in response to SCI. PI3K and downstream signaling often interacts with other cell signaling cascades, such as MAPK-Erk signaling [44], [45], which has its own repertoire of intracellular effects, presenting the possibility of unintended outcomes. Figure 9 depicts a schematic diagram of the proposed mechanism of bpV action in this study, including potential cross-talk between Erk and PI3K/Akt/mTOR signaling.

Along these lines, PTEN deletions and mutations are commonly associated with cancer, as the loss of PTEN antagonism of PI3K dysregulates the balance between cellular senescence and proliferation [53]–[55]. To minimize such concerns, short-term bpV-therapy allows strict control of timing, dosing, and withdrawal of use of the drug. In the present study, a one-week treatment regimen of bpV(pic) promoted significant long-term neuroprotective effects and functional abilities of the forelimb following treatment, and such a short therapeutic time period should have minimal impact on the threat of tumor formation compared to long-term reduction of PTEN activity or PTEN deletion. Nonetheless, further studies are necessary to investigate potential side effects of bpV therapy following SCI. In conclusion, acute administration of 400 µg/kg bpV(pic) following cervical contusive SCI afforded anatomical and functional forelimb benefits, important factors for victims suffering from cervical injuries [56]. This report is a novel step toward understanding the mechanism and benefits of bpV treatment on injured spinal tissue and associated functional outcome, and provides a foundation for future studies utilizing bpV in combinational therapies for improving anatomical and functional recovery after cervical SCI.

## Supporting Information

Figure S1 **Forelimb ability between sham, injured, and injured with bpV treatment.** Representative micrographs depicting forepaw usage while handling flavored cereal rings during the forelimb functional assessment. Sham animals demonstrate the ability to fully grasp and coordinate movement of the treat between forepaws, while injured non-treated animals primarily support the treat with the injured flexed paw. bpV-treated animals demonstrate near-sham ability to handle and manipulate the treats 6 weeks-post injury.
(TIF)

Figure S2 **GFAP labeling intensity is not significantly different 6 weeks post-SCI between treatment groups.** Relative intensity of ipsilateral GFAP intensity is similar between vehicle- and bpV-treated animal groups, suggesting bpV treatment does not result in an increased chronic glial scar formation. *n* = 4−5. Scale bar = 0.5 mm.
(TIF)

Table S1 **Forelimb Assessment Scoring Guide.**
(PPT)

Table S2 **Forelimb Assessment Score Sheet.**
(PPT)

## References

[1] Casella GT, Bunge MB, Wood PM (2006). Endothelial cell loss is not a major cause of neuronal and glial cell death following contusion injury of the spinal cord.. Exp Neurol.

[2] Tator CH, Fehlings MG (1991). Review of the secondary injury theory of acute spinal cord trauma with emphasis on vascular mechanisms.. J Neurosurg.

[3] Rami A, Kögel D (2008). Apoptosis meets autophagy-like cell death in the ischemic penumbra: Two sides of the same coin?. Autophagy.

[4] Kanno H, Ozawa H, Sekiguchi A, Itoi E (2009). Spinal cord injury induces upregulation of Beclin 1 and promotes autophagic cell death.. Neurobiol Dis.

[5] Kanno H, Ozawa H, Sekiguchi A, Yamaya S, Itoi E (2011). Induction of autophagy and autophagic cell death in damaged neural tissue after acute spinal cord injury in mice..

[6] Stokoe D, Stephens LR, Copeland T, Gaffney PR, Reese CB (1997). Dual role of phosphatidylinositol-3,4,5-trisphosphate in the activation of protein kinase B.. Science.

[7] Alessi DR, James SR, Downes CP, Holmes AB, Gaffney PR (1997). Characterization of a 3-phosphoinositide-dependent protein kinase which phosphorylates and activates protein kinase Balpha.. Curr Biol.

[8] Nave BT, Ouwens M, Withers DJ, Alessi DR, Shepherd PR (1999). Mammalian target of rapamycin is a direct target for protein kinase B: identification of a convergence point for opposing effects of insulin and amino-acid deficiency on protein translation.. Biochem J.

[9] Datta SR, Dudek H, Tao X, Masters S, Fu H (1997). Akt phosphorylation of BAD couples survival signals to the cell-intrinsic death machinery.. Cell.

[10] Cross DA, Alessi DR, Vandenheede JR, McDowell HE, Hundal HS (1994). The inhibition of glycogen synthase kinase-3 by insulin or insulin-like growth factor 1 in the rat skeletal muscle cell line L6 is blocked by wortmannin, but not by rapamycin: evidence that wortmannin blocks activation of the mitogen-activated protein kinase pathway in L6 cells between Ras and Raf.. Biochem J.

[11] Zhou BP, Liao Y, Xia W, Zou Y, Spohn B (2001). HER-2/neu induces p53 ubiquitination via Akt-mediated MDM2 phosphorylation.. Nat Cell Biol.

[12] Yu F, Sugawara T, Maier CM, Hsieh LB, Chan PH (2005). Akt/Bad signaling and motor neuron survival after spinal cord injury.. Neurobiol Dis.

[13] Noshita N, Lewén A, Sugawara T, Chan PH (2001). Evidence of phosphorylation of Akt and neuronal survival after transient focal cerebral ischemia in mice.. J Cereb Blood Flow Metab.

[14] Xu JT, Zhao X, Yaster M, Tao YX (2010). Expression and distribution of mTOR, p70S6K, 4E-BP1, and their phosphorylated counterparts in rat dorsal root ganglion and spinal cord dorsal horn.. Brain Res.

[15] Cai QY, Chen XS, Zhong SC, Luo X, Yao ZX (2009). Differential expression of PTEN in normal adult rat brain and upregulation of PTEN and p-Akt in the ischemic cerebral cortex.. Anat Rec (Hoboken).

[16] Liu K, Lu Y, Lee JK, Samara R, Willenberg R (2010). PTEN deletion enhances the regenerative ability of adult corticospinal neurons.. Nat Neurosci.

[17] Dahia PL (2000). PTEN, a unique tumor suppressor gene.. Endocr Relat Cancer.

[18] Liu C, Wu J, Xu K, Cai F, Gu J (2010). Neuroprotection by baicalein in ischemic brain injury involves PTEN/AKT pathway.. J Neurochem.

[19] Ning K, Drepper C, Valori CF, Ahsan M, Wyles M (2010). PTEN depletion rescues axonal growth defect and improves survival in SMN-deficient motor neurons.. Hum Mol Genet.

[20] Zhang QG, Wu DN, Han D, Zhang GY (2007). Critical role of PTEN in the coupling between PI3K/Akt and JNK1/2 signaling in ischemic brain injury.. FEBS Lett.

[21] Shi GD, Ouyang YP, Shi JG, Liu Y, Yuan W (2011). PTEN deletion prevents ischemic brain injury by activating the mTOR signaling pathway.. Biochem Biophys Res Commun.

[22] Nakashima S, Arnold SA, Mahoney ET, Sithu SD, Zhang YP (2008). Small-molecule protein tyrosine phosphatase inhibition as a neuroprotective treatment after spinal cord injury in adult rats.. J Neurosci.

[23] Yang P, Dankowski A, Hagg T (2007). Protein tyrosine phosphatase inhibition reduces degeneration of dopaminergic substantia nigra neurons and projections in 6-OHDA treated adult rats.. Eur J Neurosci.

[24] Schmid AC, Byrne RD, Vilar R, Woscholski R (2004). Bisperoxovanadium compounds are potent PTEN inhibitors.. FEBS Lett.

[25] Song W, Volosin M, Cragnolini AB, Hempstead BL, Friedman WJ (2010). ProNGF induces PTEN via p75NTR to suppress Trk-mediated survival signaling in brain neurons.. J Neurosci.

[26] Zhang QG, Wu DN, Han D, Zhang GY (2007). Critical role of PTEN in the coupling between PI3K/Akt and JNK1/2 signaling in ischemic brain injury.. FEBS Lett.

[27] Sury MD, Vorlet-Fawer L, Agarinis C, Yousefi S, Grandgirard D (2011). Restoration of Akt activity by the bisperoxovanadium compound bpV(pic) attenuates hippocampal apoptosis in experimental neonatal pneumococcal meningitis.. Neurobiol Dis.

[28] Cheng HC, Kim SR, Oo TF, Kareva T, Yarygina O (2011). Akt suppresses retrograde degeneration of dopaminergic axons by inhibition of macroautophagy.. J Neurosci.

[29] Gensel JC, Tovar CA, Hamers FP, Deibert RJ, Beattie MS (2006). Behavioral and histological characterization of unilateral cervical spinal cord contusion injury in rats.. J Neurotrauma.

[30] Gruner JA (1992). A monitored contusion model of spinal cord injury in the rat.. J Neurotrauma.

[31] Xu J, Fan G, Chen S, Wu Y, Xu XM (1998). Methylprednisolone inhibition of TNF-alpha expression and NF-kB activation after spinal cord injury in rats.. Brain Res Mol Brain Res.

[32] Yan P, Xu J, Li Q, Chen S, Kim GM (1999). Glucocorticoid receptor expression in the spinal cord after traumatic injury in adult rats.. J Neurosci.

[33] Yan P, Liu N, Kim GM, Xu J, Li Q (2003). Expression of the type 1 and type 2 receptors for tumor necrosis factor after traumatic spinal cord injury in adult rats.. Exp Neurol.

[34] Liu NK, Zhang YP, Titsworth WL, Jiang X, Han S (2006). A novel role of phospholipase A2 in mediating spinal cord secondary injury.. Ann Neurol.

[35] Martinez M, Brezun JM, Bonnier L, Xerri C (2009). A new rating scale for open-field evaluation of behavioral recovery after cervical spinal cord injury in rats.. J Neurotrauma.

[36] Kabeya Y, Mizushima N, Ueno T, Yamamoto A, Kirisako T (2000). LC3, a mammalian homologue of yeast Apg8p, is localized in autophagosome membranes after processing.. EMBO J.

[37] Wen S, Stolarov J, Myers MP, Su JD, Wigler MH (2001). PTEN controls tumor-induced angiogenesis.. Proc Natl Acad Sci U S A.

[38] Liu LZ, Li C, Chen Q, Jing Y, Carpenter R (2011). MiR-21 Induced Angiogenesis through AKT and ERK Activation and HIF-1á Expression.. PLoS One.

[39] Park JH, Lee JY, Shin DH, Jang KS, Kim HJ (2011). Loss of Mel-18 induces tumor angiogenesis through enhancing the activity and expression of HIF-1á mediated by the PTEN/PI3K/Akt pathway.. Oncogene.

[40] Han S, Arnold SA, Sithu SD, Mahoney ET, Geralds JT (2010). Rescuing vasculature with intravenous angiopoietin-1 and alpha v beta 3 integrin peptide is protective after spinal cord injury.. Brain.

[41] Irvine KA, Ferguson AR, Mitchell KD, Beattie SB, Beattie MS (2010). A novel method for assessing proximal and distal forelimb function in the rat: the Irvine, Beatties and Bresnahan (IBB) forelimb scale.. J Vis Exp.

[42] Harrington EP, Zhao C, Fancy SP, Kaing S, Franklin RJ (2010). Oligodendrocyte PTEN is required for myelin and axonal integrity, not remyelination.. Ann Neurol.

[43] Lu K, Liang CL, Liliang PC, Yang CH, Cho CL (2010). Inhibition of extracellular signal-regulated kinases 1/2 provides neuroprotection in spinal cord ischemia/reperfusion injury in rats: relationship with the nuclear factor-kappaB-regulated anti-apoptotic mechanisms.. J Neurochem.

[44] Lehman JA, Gomez-Cambronero J (2002). Molecular crosstalk between p70S6k and MAPK cell signaling pathways.. Biochem Biophys Res Commun.

[45] Ma L, Chen Z, Erdjument-Bromage H, Tempst P, Pandolfi PP (2005). Phosphorylation and functional inactivation of TSC2 by Erk implications for tuberous sclerosis and cancer pathogenesis.. Cell.

[46] Dümmler BA, Hauge C, Silber J, Yntema HG, Kruse LS (2005). Functional characterization of human RSK4, a new 90-kDa ribosomal S6 kinase, reveals constitutive activation in most cell types.. J Biol Chem.

[47] Codeluppi S, Svensson CI, Hefferan MP, Valencia F, Silldorff MD (2009). The Rheb-mTOR pathway is upregulated in reactive astrocytes of the injured spinal cord.. J Neurosci.

[48] Cho J, Lee SH, Seo JH, Kim HS, Ahn JG (2002). Increased expression of phosphatase and tensin homolog in reactive astrogliosis following intracerebroventricular kainic acid injection in mouse hippocampus.. Neurosci Lett.

[49] Scott RC, Schuldiner O, Neufeld TP (2004). Role and regulation of starvation-induced autophagy in the Drosophila fat body.. Dev Cell.

[50] Posner BI, Faure R, Burgess JW, Bevan AP, Lachance D (1994). Peroxovanadium compounds. A new class of potent phosphotyrosine phosphatase inhibitors which are insulin mimetics.. J Biol Chem.

[51] Lu Y, Yu Q, Liu JH, Zhang J, Wang H (2003). Src family protein-tyrosine kinases alter the function of PTEN to regulate phosphatidylinositol 3-kinase/AKT cascades.. J Biol Chem.

[52] Thomas SM, Brugge JS (1997). Cellular functions regulated by Src family kinases.. Annu Rev Cell Dev Biol.

[53] Suzuki A, de la Pompa JL, Stambolic V, Elia AJ, Sasaki T (1998). High cancer susceptibility and embryonic lethality associated with mutation of the PTEN tumor suppressor gene in mice.. Curr Biol.

[54] Dahia PL, Aguiar RC, Alberta J, Kum JB, Caron S (1999). PTEN is inversely correlated with the cell survival factor Akt/PKB and is inactivated via multiple mechanisms in haematological malignancies.. Hum Mol Genet.

[55] Stiles B, Gilman V, Khanzenzon N, Lesche R, Li A (2002). Essential role of AKT-1/protein kinase B alpha in PTEN-controlled tumorigenesis.. Mol Cell Biol.

[56] Anderson KD (2004). Targeting recovery: priorities of the spinal cord-injured population.. J Neurotrauma.

