# Supplementary figures and images for: Systemic Bisperoxovanadium Activates Akt/mTOR, Reduces Autophagy, and Enhances Recovery following Cervical Spinal Cord Injury
Source: PLoS One. 2012 Jan 10;7(1):e30012. doi: 10.1371/journal.pone.0030012 (PMC3254642; doi:10.1371/journal.pone.0030012)

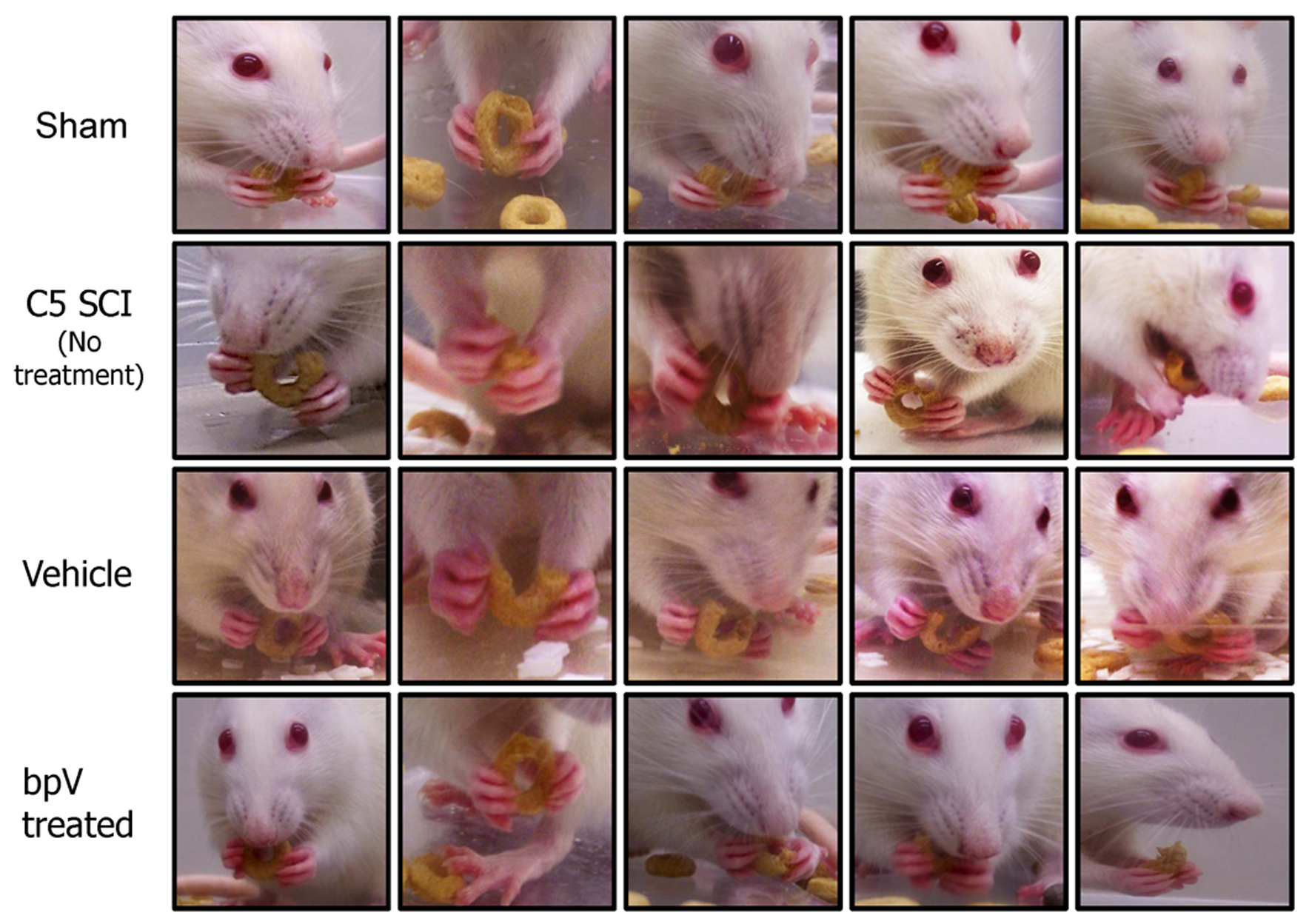

Supplement: Figure S1 — Forelimb ability between sham, injured, and injured with bpV treatment. Representative micrographs depicting forepaw usage while handling flavored cereal rings during the forelimb functional assessment. Sham animals demonstrate the ability to fully grasp and coordinate movement of the treat between forepaws, while injured non-treated animals primarily support the treat with the injured flexed paw. bpV-treated animals demonstrate near-sham ability to handle and manipulate the treats 6 weeks-post injury. (TIF) [file pone.0030012.s001.tif]

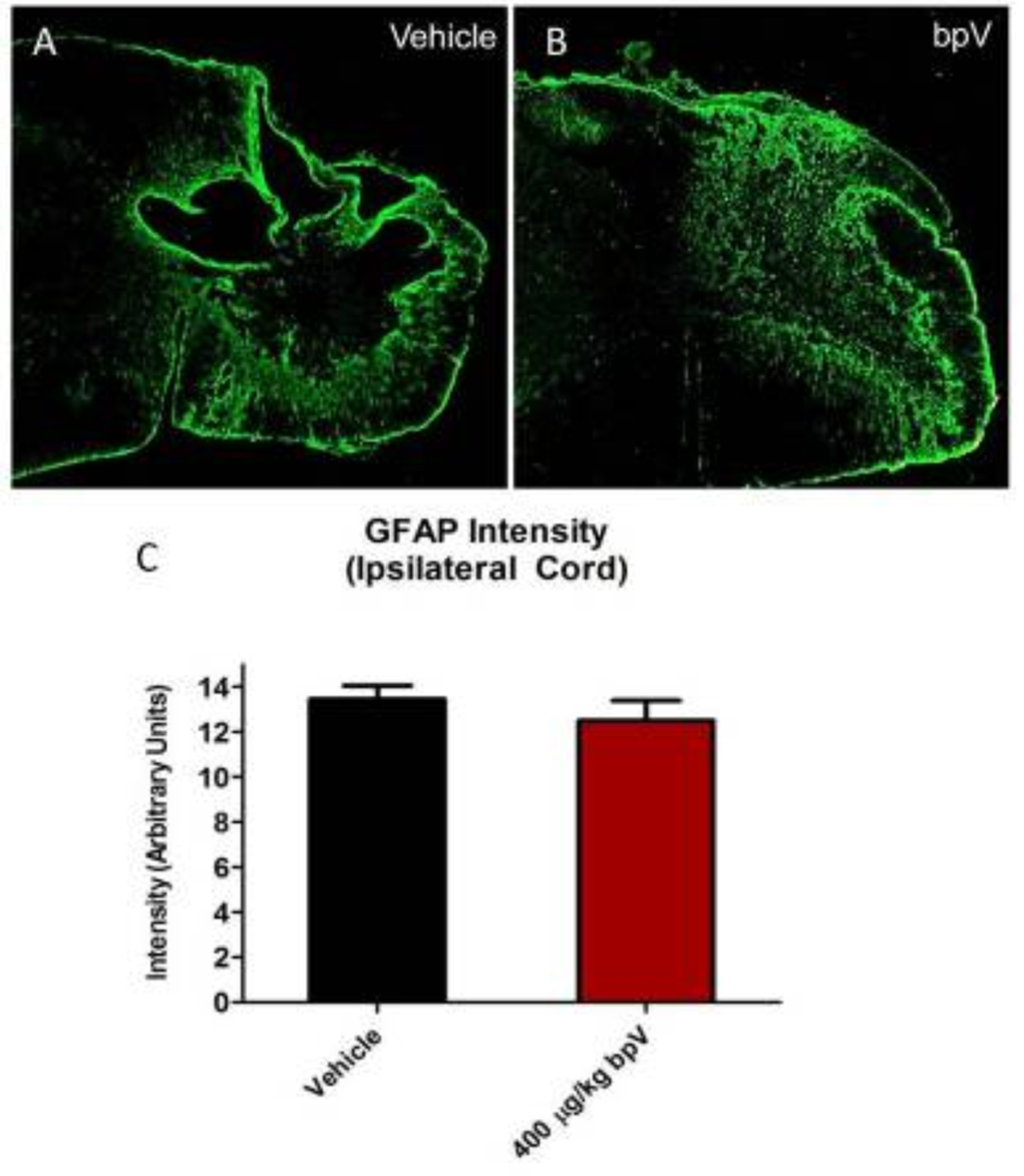

Supplement: Figure S2 — GFAP labeling intensity is not significantly different 6 weeks post-SCI between treatment groups. Relative intensity of ipsilateral GFAP intensity is similar between vehicle- and bpV-treated animal groups, suggesting bpV treatment does not result in an increased chronic glial scar formation. n = 4−5. Scale bar = 0.5 mm. (TIF) [file pone.0030012.s002.tif]

## Slide 1
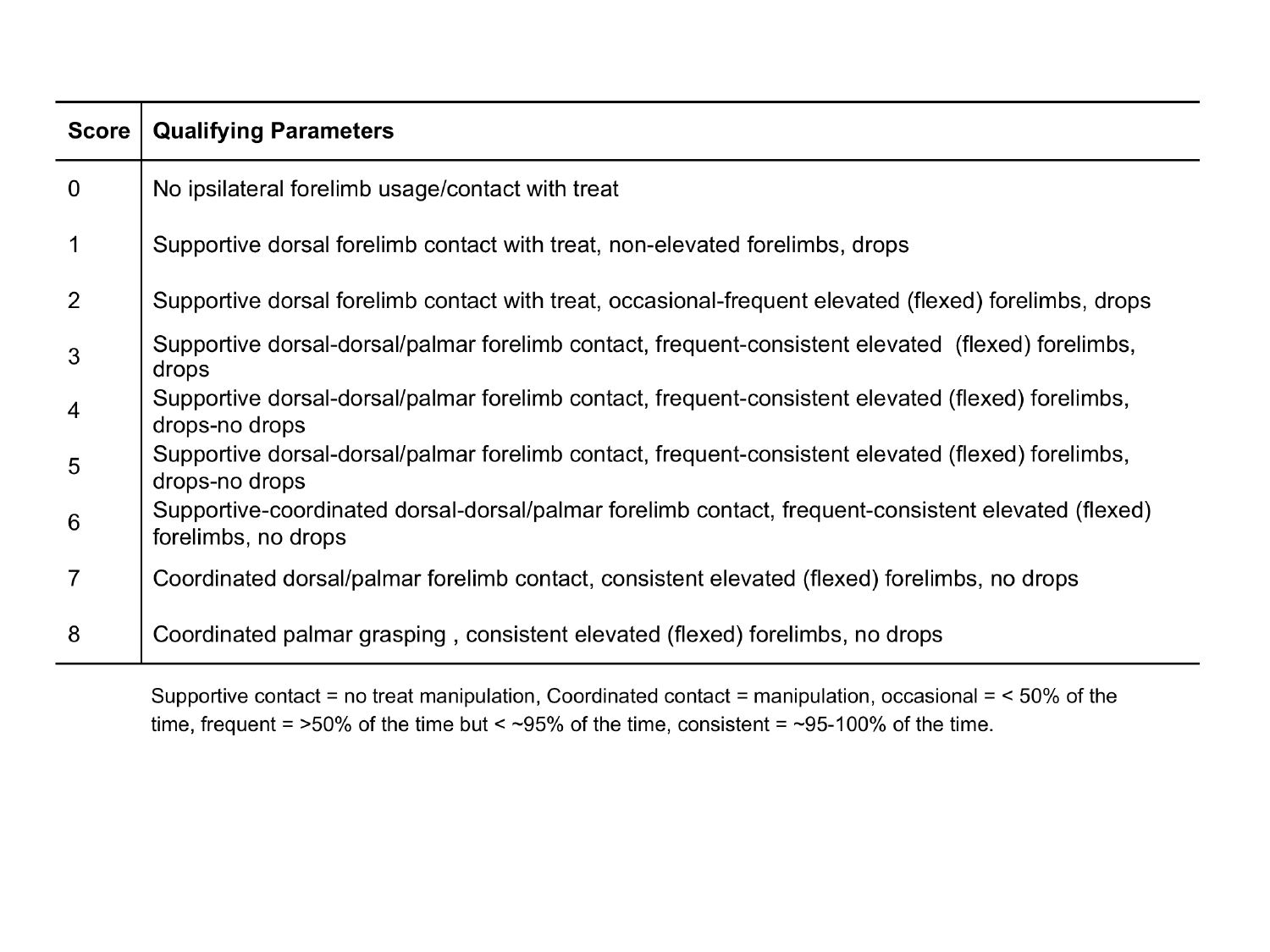

Supplement: Table S1 — Forelimb Assessment Scoring Guide. (PPT) [file pone.0030012.s003.ppt]
