# Supplementary material for: Systemic Bisperoxovanadium Activates Akt/mTOR, Reduces Autophagy, and Enhances Recovery following Cervical Spinal Cord Injury
Source: PLoS One. 2012 Jan 10;7(1):e30012. doi: 10.1371/journal.pone.0030012 (PMC3254642; doi:10.1371/journal.pone.0030012)
Supplement: Table S2 — Forelimb Assessment Score Sheet. (PPT) [file pone.0030012.s004.ppt]

## Slide 1
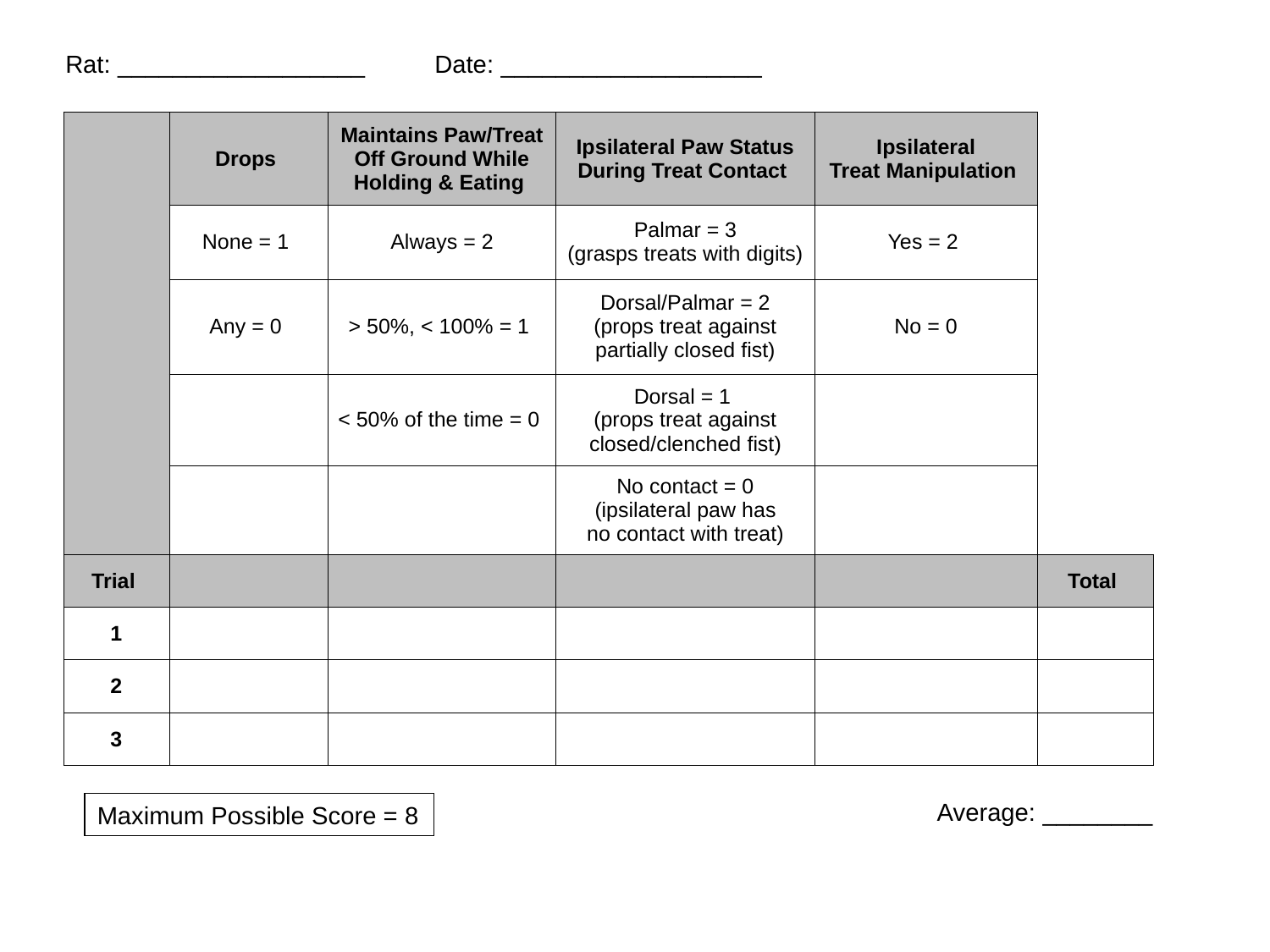

Rat: __________________ Date: ___________________
| | Drops | Maintains Paw/Treat Off Ground While Holding & Eating | Ipsilateral Paw Status During Treat Contact | Ipsilateral Treat Manipulation | |
| --- | --- | --- | --- | --- | --- |
| | None = 1 | Always = 2 | Palmar = 3 (grasps treats with digits) | Yes = 2 | |
| | Any = 0 | > 50%, < 100% = 1 | Dorsal/Palmar = 2 (props treat against partially closed fist) | No = 0 | |
| | | < 50% of the time = 0 | Dorsal = 1 (props treat against closed/clenched fist) | | |
| | | | No contact = 0 (ipsilateral paw has no contact with treat) | | |
| Trial | | | | | Total |
| 1 | | | | | |
| 2 | | | | | |
| 3 | | | | | |
 Average: ________
Maximum Possible Score = 8
